# Ribbon Synapses and Retinal Disease: Review

**DOI:** 10.3390/ijms24065090

**Published:** 2023-03-07

**Authors:** Courtney E. Frederick, David Zenisek

**Affiliations:** Department of Molecular and Cellular Physiology, Yale University School of Medicine, 333 Cedar Street, P.O. Box 208026, New Haven, CT 06510, USA

**Keywords:** ribbon synapses, retina, inherited retinal degeneration, presynaptic ribbon proteins, retinal disorders

## Abstract

Synaptic ribbons are presynaptic protein complexes that are believed to be important for the transmission of sensory information in the visual system. Ribbons are selectively associated with those synapses where graded changes in membrane potential drive continuous neurotransmitter release. Defective synaptic transmission can arise as a result of the mutagenesis of a single ribbon component. Visual diseases that stem from malfunctions in the presynaptic molecular machinery of ribbon synapses in the retina are rare. In this review, we provide an overview of synaptopathies that give rise to retinal malfunction and our present understanding of the mechanisms that underlie their pathogenesis and discuss muscular dystrophies that exhibit ribbon synapse involvement in the pathology.

## 1. Introduction

Photoreceptor and bipolar cell synapses utilize specialized multiprotein structures called synaptic ribbons to modulate neurotransmission [1,2,3]. Ribbon synapses are able to receive and process external stimuli over long periods and in response to fluctuating intensities of light. Apart from their tonic nature, they are distinguishable from conventional synapses by the presence of these multiprotein complexes with Ribeye as its unique and major component [4,5,6,7,8,9]. Although aspects of ribbon function remain unclear, ribbons serve as a scaffold for a cache of synaptic vesicles and actively contribute to vesicular release at the active zone [1,4,10,11,12,13]. Much of the scientific inquiry dedicated to ribbon synapses involve the individual contributions of the proteins that make up the ribbon, and while these studies have led to our understanding of ribbon function, the involvement of ribbon synapses in retinal diseases represents an underdeveloped area of study.

**Figure 1:**
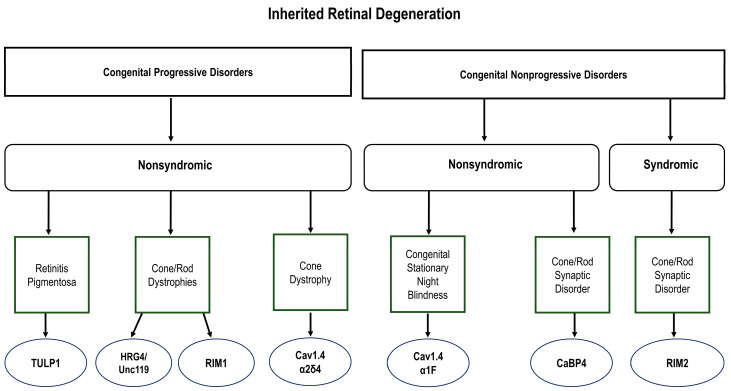
Inherited retinal degeneration (IRD) is a common form of vision disease in humans. Characterized by photoreceptor degeneration, mutations in the represented elements of the ribbon synapse induce both nonsyndromic and syndromic forms of these diseases. The clinical manifestations of the diseases caused by these dysfunctions are diverse.

Inherited retinal degeneration (IRD) is an umbrella term for those heritable diseases that produce photoreceptor dysfunction and degeneration [14]. All previously described forms of visual dysfunction arising from the mutagenesis of ribbon proteins fall under this category of disease (Figure 1). IRD is one of the leading causes of blindness, affecting 1 in 2–3000 (1/2000–3000) individuals worldwide with a subset of those diseases arising from ribbon protein involvement. Those ultimately diagnosed with IRD typically present with visual difficulties in childhood, although the age of onset, the progression of vision loss, and the severity of symptoms can vary greatly from one person to another even amongst individuals with the same mutation. It is therefore difficult to predict the time course of vision loss for any one person. The classification of IRDs can therefore be complicated by this diversity of clinical manifestations (Table 1) [14,15,16,17].

The hallmark feature of IRDs or retinal dystrophies is loss of photoreceptors and can be categorized by the rate at which this photoreceptor loss occurs. Those disorders that elicit continual photoreceptor loss are deemed ‘progressive’, while those that have a slower rate of degeneration or stabilize are classified as ‘nonprogressive’ disorders. IRDs can also be segregated by the order in which photoreceptor loss occurs—Rod/Cone dystrophies, which occur once in every 4000 live births (1/4000), and the less frequent Cone/Rod (CORD) dystrophies (1/40,000) [14,16,25]. Sufferers experience reduced visual acuity that cannot be corrected and may experience any of the following visual maladies as photoreceptor loss progresses: photophobia, tunnel vision, color blindness (dyschromatopsia), and night blindness, depending upon the type of IRD they are afflicted with and the region of the retina affected. Individuals diagnosed with progressive forms of IRD are typically rendered legally blind by mid-adulthood [16,17]. IRDs can also be roughly subdivided by the region of the retina affected: 1. macular, or central disorders; 2. central and peripheral disorders; and 3. peripheral disorders [14,15]. The genetic classification of IRDs is made complex by the number of genes that produce them; these disorders can arise from mutations in over 280 genes that vary in developmental and functional roles [14]. A single genetic mutation can yield different symptoms and alternate rates of disease progression in different individuals [15].

In addition to retinal dystrophies, the mutagenesis of other protein elements of ribbon presynaptic machinery have been shown to be associated with syndromic diseases, congenital degenerative diseases, and infectious diseases. This review focuses on diseases that affect presynaptic proteins and structures and their underlying pathogenesis and clinical manifestations. We also provide an overview of muscular dystrophies that have concomitant retinal ribbon synapse involvement.

## 2. Nonsyndromic Congenital Progressive Retinal Disorders

### 2.1. Human Retinal Gene 4 (HRG4/Unc119/Munc119)

**Figure 2:**
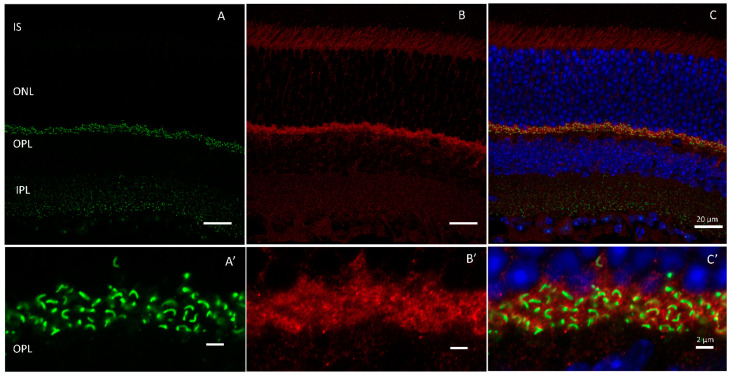
HRG4/Unc119 is expressed in the outer plexiform layer of murine retinas. Immunolabeling of ribbon synapses with (**A**) Ribeye (green) and (**B**) HRG4/Unc119 (red). (**C**) merged. Outer plexiform layer (**A’**–**C’**). Scale bar = 20 µm in (**A**–**C**) and 2 µm in (**A’**–**C’**).

HRG4/Unc119 was first isolated as part of a study undertaken to identify novel retinal genes [26]. As the fourth gene cloned from a subtractive library screen for retinal enriched cDNAs, it was given the moniker ’human retinal gene 4‘ or HRG4 [26,27]. Subsequently, HRG4 was determined to be the human orthologue of the *Caenorhabditis elegans* (*C. elegans)* gene uncoordinated 119 (*unc-119)* [28,29]. Unc119 is expressed in the retina and is highly enriched in photoreceptor synapses (Figure 2) where it has been shown to bind to calcium binding protein 4 (CaBP4), Ribeye, and ADP-ribosylation factor (ARF)-like protein 2 (ARL2) [28,30,31,32,33]. In the retina, it is hypothesized to participate in vesicular trafficking at photoreceptor terminals [28,30,31].

HRG4/Unc119 was mapped to chromosome 17q11.2 within the locus previously associated with inherited retinal disease [34]. Equipped with this information, 138 human patients previously diagnosed with retinal degeneration were then screened for mutations in the HRG4 gene. One patient diagnosed with late-onset retinal degeneration harboring a heterozygous mutation (A57T) in HRG4 resulting in a premature stop codon was identified in that screen [35]. In order to characterize the nature of the pathogenicity of the A57T mutation, a transgenic mouse model expressing the truncated protein in rods was created [35]. Characterization of the mouse model revealed a decrease in the expression of several ribbon proteins and a significant reduction in the size of the inner plexiform layer, as demonstrated through immunohistochemical and Western blot analysis [31].

Further analyses on the transgenic mouse were conducted by electroretinogram (ERG). ERGs measure the electrical signals retinas generated in response to light that are represented by a series of waveforms. The first, called the a-wave, is produced by photoreceptors and the second ‘b-wave’ arises from the electrical responses of bipolar cells [36]. As with patients, mice harboring the heterozygous mutation displayed age-related photoreceptor degeneration and abnormalities in the b-wave on an ERG indicating a dysfunction in synaptic transmission [35]. Subsequently, an *unc119* knockout mouse was generated and demonstrated an accelerated rate of photoreceptor degeneration together with a reduction in both the ERG a- and b-waves [37].

Saturation binding assays performed with recombinant full length or the truncated HRG119 produced by the patient mutation and native ARL2 obtained from retinal extracts led investigators to propose a molecular mechanism underpinning the photoreceptor loss in this disease [38]. They hypothesized that the truncated protein produced in the disease state has a stronger affinity to ARL2, but that their interaction is probably nonfunctional [38]. As a result, they posit that the ARL2 is sequestered and that this sequestration negatively impacts downstream processes. Namely, the interactions of ARL2 with its binding partners in the mitochondria can no longer occur, but instead trigger an apoptotic pathway that ultimately destroys the entire photoreceptor, resulting in vision loss [38].

### 2.2. Regulating Synaptic Membrane Exocytosis Protein 1/Rab3 Interacting Molecule 1 (Rim1)

In 2003, a cone/rod dystrophy in *RIMS1* was mapped to chromosome 6q14, a region near several previously identified loci for retinal disorders [39,40]. The resultant mutation, Rim1 (R844H), was discovered in several generations of a British family who reported having visual difficulties that began as deficiencies in color vision and progressed to reduced visual clarity, especially in bright light and in one instance dim light as well [39,41]. It was reported that onset of initial symptoms occurred between the ages of 20 and 40 and examination of the fundus revealed macular changes that ranged from mild disorganization of the retinal pigment epithelium (RPE) to severe atrophy [39,42]. Pattern electroretinography (PERG), a test employed to measure central retinal function, revealed macular impairment [39,42,43]. ERGs performed on the affected members revealed diminished photoreceptor function while the b-wave appeared unaffected, signaling that the source of dysfunction is not at the level of synaptic transmission [42]. When tracked, ERG testing also showed a decline in rod function over time, characteristic of cone/rod dystrophies.

Rim1 was discovered in a search for the effector of Rab3 from yeast two-hybrid screening of a rat brain library [44]. The protein features two calcium-binding domains, C2A and C2B. Rim1 has also been reported to interact with synaptotagmin, SNAP-25, and the α1D subunit of calcium channels [39,45,46]. Pinpointing the exact localization of Rim protein expression in the retina has been complicated by the number of splice isoforms produced by the *RIMS1* gene and the specificity of the antibodies used to localize its retinal expression. Early immunohistochemical studies reported outer plexiform expression of Rim1 and Rim2 at photoreceptor terminals where Rim1 was proposed to interact directly with Munc-13 and Rab3 [2,44]. Rab proteins are a large family of GTPases that regulate the vesicular transport between cellular compartments [47]. Multiple Rabs are expressed throughout the nervous system with Rab3 isoforms localized to synaptic vesicles (Rab3a, Rab3b, Rab3c, and Rab3d) and important for normal calcium mediated vesicular exocytosis [48]. Rab3 isoforms appear to function interchangeably at conventional synapses, but rab3a appears to be the dominant isoform at retinal ribbon synapses [48,49].

Multiple lines of evidence gathered from Rim1 studies conducted in mice raise several questions. First and foremost, it has been shown that Rim1 is likely absent from photoreceptors in the mouse at the protein and transcript level [19]. Moreover, the R855H mutation in RIM1 which resides in the C2A domain of the protein does not influence its ability to bind Ca-channels or change the voltage-dependence and kinetic properties of Ca-currents in cell lines overexpressing RIM1 and L or P/Q type calcium channel constructs [39,46]. Rim1 deficient mice fail to show severe defects in ERGs or photoreceptor synaptic function, thus the apparent conflict in the data remain to be resolved [19]. It is possible that a phenotype may only arise in older animals and an examination of the aged mouse might be clarifying [19,50]. Alternatively, either dysfunctional Rim1 in the scarce quantities expressed in photoreceptors might be sufficient to produce a cone/rod dystrophy or it is able to exert its effects on photoreceptors from other cell types. Additionally, the knockout mouse may not serve as an appropriate model for CORD7, as human retinas may differ in their Rim expression from the mouse or the mutation may cause pathologies that are not captured by deletion of the gene. Alternatively, Rim1 mutations may cosegregate with another disease genotype that has gone undetected.

### 2.3. Tubby-like Protein-1 (Tulp1)

Retinitis Pigmentosa (RP) (1:4000) is a clinically and genetically diverse subset of IRDs characterized by progressive dysfunction of the rods prior to degeneration of cone photoreceptors [25]. Visual symptoms typically emerge in the form of night blindness and progressive visual field loss. Mutagenesis of the presynaptic ribbon protein, Tulp1, has been identified as the root of RP affecting the peripheral retina in a subset of patients [51]. Tulp1 belongs to the Tubby gene family, which consists of multiple members that have been implicated in retinal disease. The founding family member, tubby was first identified in a mouse line that arose from spontaneous mutation in the Jackson Lab colony [52,53,54,55]. This was described as having a propensity for obesity, compromised fertility, and a combination of hearing and vision loss over time [52]. The gene responsible for the aforementioned phenotypes, *tub*, was mapped to chromosome 7 and the mouse was given the moniker ‘tubby’ [52,53,54,55]. The sensory defects in the tubby mouse were found to be a result of cochlear and retinal degeneration following a pattern akin to Usher Syndrome. Classifying it as an Usher model was not straightforward, however, because concomitant obesity made it suitable for a model for Alstrom syndrome or Bardet–Biedl syndrome [55].

**Figure 3:**
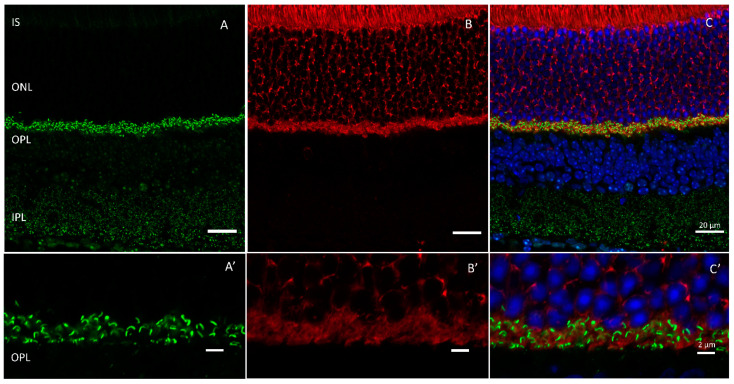
Tulp1 is expressed through retinal photoreceptors where it interacts with Ribeye in the outer plexiform layer. Wildtype mouse retinas labeled with antibodies against (**A**) Ribeye (green) and (**B**) Tulp1 (red). Outer plexiform layer shown in (**A’**–**C’**). Scale bar = 20 µm in (**A**–**C**) and 2 µm in (**A’**–**C’**).

Tubby belongs to the ’tubby-like’ family of proteins (Tulps) that include TUB, Tulp1, Tulp2, and Tulp3 [56]. All isoforms share a conserved phosphoinositol-4,5-bisphosphate (PIP2) binding C-terminal domain. Tubby and Tulp1 are highly expressed in the retina [51,57]. Of the Tulp isoforms identified, only Tulp1 is located exclusively in the human retina where it is expressed throughout the photoreceptors (Figure 3), including the synaptic terminals, but excluded from the inner plexiform layer [58].

*Tulp1* maps to chromosome 6p21.3, which is positioned within the region of other known retinitis pigmentosa disease loci. Therefore, although tubby mutations in the mouse led to an array of symptoms that were associated with syndromic disease, Tulp1 was explored in humans as an RP causative gene [51,56,57,59,60]. To this end, several groups conducted screens on individuals who had been diagnosed with RP in a search for mutations in Tulp1 [51,61,62]. From these studies, several individuals bearing missense mutations in Tulp1 were identified. Although some of the individuals identified suffered from extremely compromised photoreceptor function, none of them presented with concomitant obesity or hearing loss [51]. Additionally, although the mutations that produced the disease state varied in affected individuals, it was found that the same conserved c-terminal ‘tubby’ region of the protein was disrupted in all instances [51,61,62].

More recently, a duplication of two amino acids (FA531–532dup) in exon 15 of Tulp1 was determined to cause an aggressive form of IRD: Leber congenital amaurosis (LCA), or, early-onset retinal degeneration (EORD), in an Algerian family [63]. Patients, who were diagnosed by age 3, all suffered from night blindness, nystagmus, extremely poor visual acuity, and in some cases color blindness.

Mice lacking Tulp1 experience photoreceptor degeneration without hearing loss or obesity, supporting its underpinning as an IRD causative agent [18]. Further, the mouse demonstrated synaptic disruption [64]. At the ribbon synapse, tubby-like protein-1 directly interacts with Kif3a, dynamin-1, and Ribeye and colocalizes with clathrin [58,65,66]. This association with key vesicular proteins suggests that it plays a vital role in trafficking [58]. When young mice lacking Tulp-1 were analyzed by ERG, there was an overall reduction in the b-wave, indicating synaptic transmission dysfunction [58]. Immunohistochemical assays to pinpoint the specific mechanism of defect in the Tulp1 null mice showed that the endocytic proteins located around the active zone of ribbon synapses were dramatically diminished while active zone proteins remained, suggesting a key role for Tulp1 in vesicular recycling [65].

## 3. Nonsyndromic Congenital Nonprogressive Retinal Disorders

### 3.1. Calcium Channels

**Figure 4:**
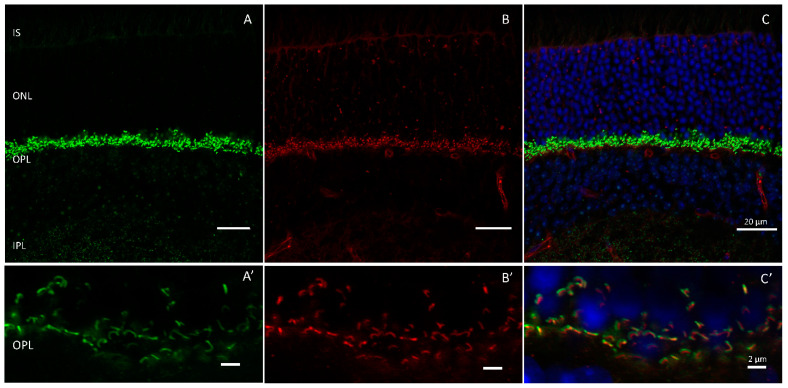
Calcium channels are localized to the outer plexiform layer where they are closely associated with ribbons at the synapse. Shown is antibody staining of an adult wildtype mouse retina labeled with (**A**) Ribeye (green) and (**B**) Calcium channel pore forming subunit, α1F (red). (**C**) merged. Outer plexiform layer shown in (**A’**–**C’**). Scale bar = 20 µm in (**A**–**C**) and 2 µm in (**A’**–**C’**).

Presynaptic voltage-gated calcium (Ca^2+^) channels are located in close proximity to ribbons at the synapse where they initiate neurotransmission when activated [20,67]. The general nomenclature, classification, function, and properties of calcium channels are reviewed in [67,68,69]. In the retina, Ca_v_1.4, an L-type voltage-gated calcium channel, drives release from rod photoreceptors. Cav1.4 comprises an alpha 1F (α1F) pore-forming subunit (*CACNA1F*) (Figure 4) and three additional subunits (α_2_δ_4_, β_2_, and γ) that combine to form the functional channel [21,68]. Mutations in individual subunits of Ca_v_1.4 have been shown to cause retinal pathogenesis.

#### 3.1.1. α1. F Subunit (CACNA1F)

Congenital stationary night blindness (CSNB) (1:40,000) is characterized by impaired vision under dim lighting conditions, reduced visual acuity, strabismus, nystagmus, and photophobia [39]. CSNB is broadly divided into two categories, X-linked and autosomal recessive, both of which display similar ERG characteristics: a near-normal a-wave and a substantially reduced b-wave under scotopic conditions. The X-linked type of CSNB is further subdivided into complete CSNB (cCSNB or CSNB1) and incomplete CSNB (CSNB2 or iCSNB). Complete CSNB has been mapped to chromosome Xp11.4 and the defects causing this disorder are located on elements of the postsynapse in ON-type retinal bipolar cells [70,71,72,73,74].

Hundreds of mutations in the pore-forming α1F subunit of the Cav1.4 channel have been revealed as the causative defects in people diagnosed with iCSNB [75]. Mapped to chromosome Xp11.23, incomplete CSNB produces mild to moderate nearsightedness, farsightedness, and ERGs that reveal defects at the synapse in patients [75,76]. There is a large degree of variability in the clinical presentation of persons diagnosed with iCSNB and many do not experience night blindness [77].

Encoded by the gene *CACNA1F,* mutations in the pore have been uncovered in every region of the subunit and include missense and truncation mutations that most often render it nonfunctional, although decreased expression mutations and a gain-of-function mutation has also been reported [75,76,77,78,79,80,81,82,83]. This mutagenic variability may be responsible for the inconsistency of reported symptoms borne by sufferers. In the case of loss of function mutations, the disease state arises from inefficient or absent channel gating leading to defective synaptic transmission. To understand the mechanism underlying the gain-of-function mutation (I745T), a mouse model of the disease was created [84]. In mature mice, mutant channels localized to the ribbon, but the number of ribbons throughout the synaptic layer was decreased and cone terminals were observed to contain floating ribbons. Additionally, the overall morphology of the properly localized ribbons was atypical. Recordings made from the mouse revealed reduced amplitude and an increase in delay of the b-wave indicating a disruption of synaptic transmission. Additionally, there was observable degeneration of the photoreceptor layer in the mouse similar to that of rod/cone dystrophies, which contrasted with loss of function mutants that have a more equitable loss of rod-to-cone ratio [84]. This marked photoreceptor loss in the mouse also contrasts with clinical findings observed in the affected family whose visual loss, although severe, was nonprogressive, presumably reflecting a species difference phenotype [83].

#### 3.1.2. α2δ4. Subunit (CACNA2D4)

Several mutations have been identified in the α_2_δ_4_ subunit of patients suffering from what has now been identified as a rare, recessive, retinal cone dystrophy [85,86,87]. Individuals diagnosed with this disorder suffer from mildly reduced visual acuity with onset in mid-adulthood, early-onset photophobia, pigmentary deposits in the fovea [86]. Full-field ERGs obtained from the patients showed preserved, albeit subnormal rod responses. An examination of the cone responses, obtained from ERGs via photopic flash recordings, uncovered undetectable cone responses. Investigators report that the ERG results are indicative of CSNB, with two crucial differences: lack of night blindness, and the progressive nature of the disease experienced by the study subjects [86].

*CACNA2D4* encodes the α_2_δ_4_ subunit of Cav1.4 as a single product. The α_2_ domain is an extracellular glycosylated subunit and interacts the most with the α_1_ subunit, whereas the δ_4_ subunit has a single transmembrane region and anchors the protein to the plasma membrane. A mouse model with spontaneous mutation in α_2_δ4, 2367insC, which led to a premature stop codon and a truncation of the subunit, has been studied in an effort to determine the human disease mechanism [88]. The retinas of these animals had reduced *Cacna2d4* mRNA expression and rod degeneration, and when subjected to ERG testing, they displayed a significant reduction in the scotopic a-wave and a significant loss of the b-wave.

### 3.2. CaBP4

CaBP4 is one member of a protein subfamily that all bear characteristic calcium-binding helix-loop-helix domains, or ‘EF-hands’ [89]. In the retina, CaBP4 is expressed solely in photoreceptors with high expression in the synaptic terminals where it has been demonstrated to interact with the alpha-1 F subunit of calcium channels and Unc119 [33]. In 2006, 10 patients diagnosed with congenital stationary night blindness of unknown genetic origin were screened to determine the cause of their afflictions [90]. Analysis revealed these individuals carried dual mutations in the *cabp4* gene [90]. The patient mutations were predicted to produce one frameshift (Glu267fs) that would extend the length of the protein by approximately 100 amino acids, and a point mutation (370C→T) [90]. Because the frameshift is predicted to lie downstream from the EF hand motifs, it was thought to be unlikely to directly impair calcium binding. Improper folding as a result of the frameshift could obstruct the availability of the EF-hand for binding, leading to disease state. The C370T mutation, on the other hand, appears to result in an overall reduction in total CaBP4 protein expressed.

A study conducted by Littnik et al. (2009) reported the premature truncation of CaBP4 in a family that resulted in the deletion of two functional EF hands, thereby severely limiting its ability to bind calcium [91]. They considered these patients as having a newly described disorder however, primarily because patients who bore CaBP4 mutations did not experience night blindness. Instead, they proposed that the visual difficulties experienced be given the label cone rod synaptic disorder (CRSD) [91].

The investigations that uncovered human *cabp4* mutations reported varied phenotypes [90,91,92,93]. In order to resolve the clinical diagnosis of individuals with CABP4 mutations, a retrospective analysis was conducted, and it was found that all but one of the patients shared similar clinical features, and none included night blindness [93]. As a result, it was agreed that persons identified with mutations in *cabp4* be characterized as having the newly described disorder (CRSD), but this designation has not been uniformly adopted [91,94].

In the mouse, absence of CaBP4 appears to lead to a reduction in Unc119 and null mice display fewer photoreceptor terminals, and consequently, synaptic ribbons than wildtype mice [95]. Further, the synaptic layers in the retinas of these mice are disorganized. The remaining ribbons have been observed to be ectopically expressed, and there is a decrease in the overall thickness of the outer plexiform layer [22,33,95]. ERGs in cabp4 null mice revealed reduced b-waves as compared to wildtype, signaling synaptic disruption that was validated by single cell recordings from bipolar cells [95]. Taken together, these data fit the idea that the CaBP4 mutations that produce disease induce a synaptic disorder.

## 4. Syndromic Congenital Nonprogressive Retinal Disorders

### Regulating Synaptic Membrane Exocytosis 2/Rab3 Interacting Molecule 2 (Rim2)

Recently, *RIMS2* was identified as the origin of a congenital IRD [96]. In 2020, a group of individuals afflicted with visual disorders categorized as early onset IRD without a clear diagnosis were subjected to genomic screening in an effort to clarify their diagnoses. The subjects all suffered from poor visual acuity, developmental disabilities, and, in one instance, poor glucose regulation [96]. In addition, at least one patient displayed inner retinal thinning upon examination. Genomic analysis revealed that splicing of *RIMS2* resulted in a premature stop in the protein which, when homozygous, led to the disorder. Prior to testing, the group of seven unrelated individuals had been classified as having LCA or CSNB. ERG analysis on the study subjects revealed that two displayed a normal a-wave, but a reduced b-wave [96]. Patients also displayed light responses representative of malfunctions in cone bipolar cells [96]. Further examination revealed inner retinal thinning and disruption of the fovea [96].

**Figure 5:**
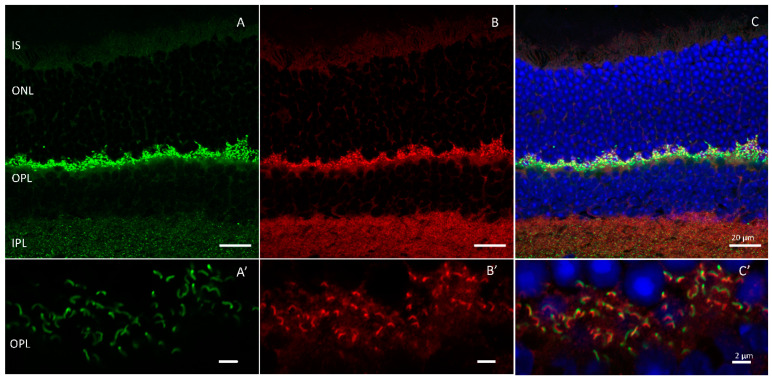
Rim2 is expressed at the ribbon synapses of the OPL and the IPL in the mouse retina. Shown is antibody staining of an adult wildtype mouse retina labeled with (**A**) Ribeye (green) and (**B**) Rim2 (red). (**C**) merged. Outer plexiform layer shown in (**A’**–**C’**). Scale bar = 20 µm in (**A**–**C**) and 2 µm in (**A’**–**C’**).

Rim2, among other functions, regulates exocytosis through the modulation of calcium channel density and localization in conventional synapses [19,96,97,98]. At ribbon synapses, it binds indirectly to L-type calcium channels through an association with rim binding proteins (RBPs) and via this interaction, it serves the same task in regulating channel location and density with respect to L-type voltage-gated calcium channels [97,98]. Immunohistochemical data collected from human tissue revealed Rim2α to be expressed in the retinal OPL, brain, and pancreas islet cells [96]. The mechanism by which truncated Rim2 leads to disease is at present unclear. At mouse photoreceptor terminals, Rim2 (Figure 5) was determined to bind only weakly to Rab3a, if at all, favoring a rab-independent mechanism [19]. Null mouse ERGs revealed normal a-waves and a slightly reduced b-wave, which suggests synaptic disruption and trends consistently with human phenotype albeit not in severity. This suggests that perhaps its deleterious effects arise from the disruption of calcium channel-mediated release at the synapses [50]. All told, the mouse phenotype appears to be subtler than the human Rim2 CRSD [50,96].

Nevertheless, taking the ERG data, Rim2 protein expression information, and the constellation of symptoms together, the Rim2 variants borne by these individuals ultimately produced a syndromic CRSD. This appears to be the first description of syndromic, nonprogressive, congenital retinal degeneration.

## 5. Inherited Syndromic Diseases with Retinal Dysfunction: Muscular Dystrophy

**Figure 6:**
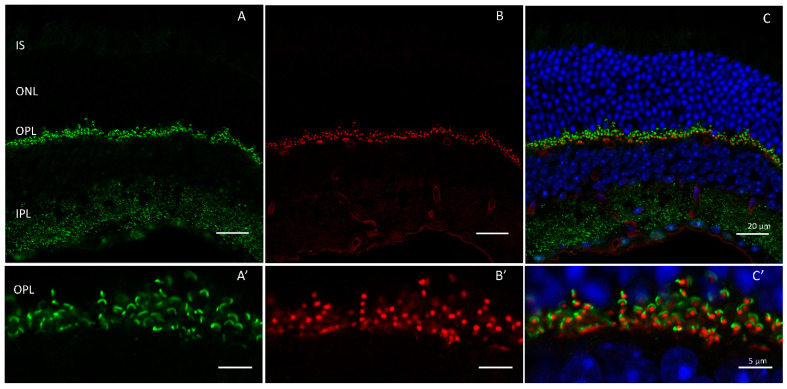
Dystrophin expression in the mouse retina is localized to the outer plexiform layer of the retina where it complexes with dystroglycan. Shown is antibody staining of an adult wildtype mouse retina labeled with (**A**) Ribeye (green) and (**B**) dystrophin (red). (**C**). Merged. Outer plexiform layer shown in (**A’**–**C’**). Scale bar = 20 µm in (**A**–**C**) and 5µm in (**A’**–**C’**).

Muscular dystrophy (MD) is an X-linked disorder characterized by progressive muscle weakening and joint hyperflexibility resulting in severely reduced mobility in early childhood and death by middle age. In addition to progressive muscle wasting, a large subset of MD sufferers also experience cognitive delays and visual dysfunction substantiated by negative ERGs [99,100,101,102,103,104,105,106]. There are multiple types of the disorder, but the most commonly known are Duchenne muscular dystrophy (DMD) (1/3500) and the less severe Becker muscular dystrophy (BMD) (1/30000), which are both X-linked disorders [107,108]. Many of the proteins that comprise the ribbon are also present at conventional nonribbon-type synapses; therefore, dysfunction of proteins at conventional synapses can be observed to have deleterious consequences in visual processing [7,8,9]. Both DMD and BMD arise because of mutations in dystrophin, a component of the dystrophin–glycoprotein complex (DGC), which is localized at neuromuscular junction postsynapses but expressed at the presynapses of photoreceptors (Figure 6) [23,109].

**Figure 7:**
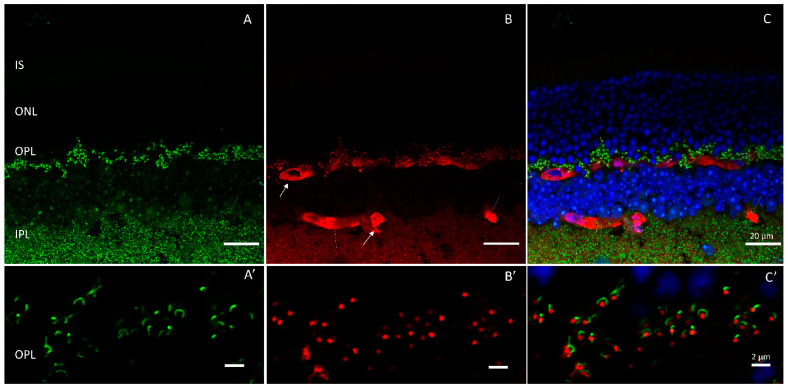
Dystroglycan is localized to the outer plexiform layer of the retina. Shown is antibody staining of an adult wildtype mouse retina labeled with (**A**) Ribeye (green) and (**B**) dystroglycan (red). (**C**) merged. Outer plexiform layer shown in (**A’**–**C’**). Retinal vasculature (*arrows*). Scale bar = 20 µm in (**A**–**C**) and 2 µm in (**A**’–**C**’).

A heterogeneous protein structure that connects muscle tissue to its surrounding matrix providing stability, the DGC facilitates force transduction and protection from damage induced by contraction [107,109,110,111]. In addition to dystrophin, the DGC is made up of at least nine other proteins, including dystroglycan (DG) (Figure 7) that connects dystrophin to the extracellular matrix [112].

Alternative forms of MD can arise as a result of mutations in other DGC proteins. Muscle, eye–brain disease (MEB) and Walker–Warburg syndrome (WWS), for example, both result from mutations in the O-linked mannose b1,2-N-acetylglucosaminyltransferase 1 (POMGnT1) gene [113]. POMGnT1 is responsible for glycosylating the alpha-subunit of dystroglycan (α-dystroglycan), a protein modification necessary for the interaction between dystroglycan and the extracellular matrix. In these forms of MD, POMGnT1 is rendered nonfunctional and α-dystroglycan is severely under-glycosylated. Likewise, Fukuyama congenital muscular dystrophy (FCMD) is also the result of glycotransferase deficiency effecting α-dystroglycan; in this case, however, the deleterious mutation occurs in the *fukutin* gene [114,115]. All of these ‘dystroglycanopathies’ are characterized by brain malformations and extreme muscle weakness [113,114]. Visual maladies that are not associated with synaptic defects have also been documented in MD patients including, but not limited to myopia, glaucoma, abnormally shaped eyes, and cataracts [116]. In the retina, the DGC is expressed at photoreceptor ribbon synapses where the protein pikachurin is also localized [24,109].

### Pikachurin

Orthodenticle homeobox 2 (Otx2) is a transcription factor, encoded by the otx2 gene, that regulates photoreceptor development [117]. An otx2 null mouse was generated to explore the function of Otx2 in the retina and subsequently used to conduct a screen to find novel genes that served to regulate photoreceptor development [24]. Microarray analysis was performed comparing retinal genes in otx2 knockout and wildtype retinas. From this screen, a protein named pikachurin was identified and isolated [24]. Pikachurin, or agrin-like (AGRINL) and EGF-like and fibronectin type-III and laminin G-like (LG) domain-containing protein (EGFLAM) is encoded by the *EGFLAM* gene. Pikachurin knockout mice revealed no gross morphological defects, but closer scrutiny of the retinal architecture through immunohistochemistry and electron microscopy revealed that photoreceptor terminals and bipolar dendrites were no longer closely aligned to each other [24]. ERG recordings demonstrated an abnormal b-wave and attenuated optokinetic responses consistent with synaptic disruption [24]. Pull-down and solid phase binding assays using recombinant pikachurin LG domains and α dystroglycan revealed that LG domains −2 and −3 of pikachurin are required for the interaction between the dystroglycan and pikachurin [118].

Mouse models of dystroglycanopathies were subsequently used to validate the findings obtained from recombinant protein binding assays. The first dystroglycanopathy mouse model, Large^myd^, bears a mutation in the gene *myd. Myd* encodes the protein Large, a glycosyltransferase [119]. In the absence of Large, α-dystroglycan is hypoglycosylated, resulting in low expression levels of the protein. The second mouse model lacks PomGnT1 [118]. Through immunoprecipitation assays conducted with recombinant pikachurin and lysates from the retinas of the genetically altered mice, it was determined that the Large-dependent modification of α-dystroglycan is necessary for pikachurin binding. Likewise, pikachurin binding to α-dystroglycan in the POMGnT1 mouse was dramatically reduced. Further, immunohistochemistry on retinal sections obtained from the Large^myd^ and PomGnT1 mice revealed that pikachurin was no longer expressed at the ribbon synapse. Thus, the glycosylation of α-dystroglycan appears to be a necessary requirement for the pikachurin–α-dystroglycan interaction and ultimately its localization to the ribbon.

Building upon these experiments, investigators used a tissue-specific dystroglycan knockout mouse (DG CKO) that lacks dystroglycan only in photoreceptors [120]. Similarly, pikachurin expression was reduced, and a prolonged ERG b-wave was observed and in addition, bipolar/photoreceptor cell contacts were disrupted [120]. Thus, both models, which have dystroglycan molecules rendered incapable of binding to pikachurin, exhibit indications that these disruptions cause the pathology observed in some types of muscular dystrophy.

## 6. Conclusions

Previous studies conducted in vertebrate and invertebrate models have contributed to our understanding of the function of synaptic ribbons [2,4,121,122,123,124]. The identities of members of the ribbon complex and their extended contacts at the synapse have been identified making targeted investigation of these proteins possible, but our present understanding of the breadth of ribbon involvement in retinal disease may well be in its infancy [5,125]. The visual diseases presented in this review represent a discussion of known disorders arising from the dysfunction in members of the ribbon presynapse. Continued study using animal models of disease will provide the basis for the development of therapeutic treatments for not only visual disorders, but also neurodegenerative diseases [126,127]. Despite the prevalence of IRDs, there is an overwhelming lack of accurate genotyping data accompanying clinical diagnoses [126,128,129,130]. This information is critical for improving diagnoses and represents an area that would benefit from significant scientific focus. Accurate genotypic diagnoses would contribute to more accurate predictions concerning the clinical course of disease in affected individuals and allow affected individuals license to accommodate impending visual limitations.

## Figures and Tables

**Table 1:** Summary of retinal diseases caused by dysfunction of proteins localized to the ribbon presynapse.

Protein	Gene	Disease	Phenotype	Antibody	Source
HRG4/Unc119	*UNC119*	Cone Rod Dystrophy	Poor night vision, defective color vision, photophobia, reduced visual acuity, myopia, macular atrophy, and pericentral ring scotomas. Late onset retinal degeneration.	13065–1-AP	Proteintech
Tulp1	*TULP1*	Retinitis Pigmentosa	Nystagmus (involuntary eye movement), hemeralopia (day blindness), mild myopia, low visual acuity, and nyctalopia (night blindness). Loss of mid-peripheral visual field followed by peripheral field loss and central vision with disease progression.	Custom [18]	Gift from Dr. Stephanie Hagstrom
Rim1	*RIMS1*	Cone Rod Dystrophy	Reduced visual clarity, especially in bright light. Disorganization of the retinal pigment epithelium (RPE). Macular disorganization and impairment.	140–023 [19]	Synaptic systems not represented
Cav1.4 α1.F	*CACNA1F*	Congenital Stationary Night Blindness	Mild to moderate nearsightedness, farsightedness, and nystagmus. Night blindness may or may not be experienced.	Custom [20]	Gift from Dr. Amy Lee
Cav1.4 α_2_δ_4_	*CACNA2D*	Cone Dystrophy	Mildly reduced visual acuity with onset in mid-adulthood. Early-onset photophobia. Foveal pigmentary deposits.	HPA031952 [21]	(Sigma) Not represented
CaBP4	*cabp4*	Cone Rod Synaptic Disorder	Nystagmus, stable low vision, photophobia, and a normal or near-normal fundus appearance.	Custom [22]	unavailable
Rim2	*RIMS2*	Syndromic Cone Rod Synaptic Disorder	Poor visual acuity. Developmental disabilities. Poor glucose regulation. Early onset retinal degeneration.	140–103 [19]	Synaptic Systems
Dystroglycan	*DAG1*	Muscular Dystrophy	Electronegative ERG not associated with ocular pathology.	11017–1-AP	Proteintech
Dystrophin	*DMD*	Muscular Dystrophy		AB15277–1006 [23]	Abcam
Pikachurin	*EGFLAM*	Muscular Dystrophy		011–22631 [24]	Wako Chemicals unavailable

## Data Availability

Data sharing is not applicable to this article.
